# Relational quality and uncertainty in common pool water management: an exploratory lab experiment

**DOI:** 10.1038/s41598-021-94517-6

**Published:** 2021-07-26

**Authors:** Marcela Brugnach, Sander de Waard, Dimitri Dubois, Stefano Farolfi

**Affiliations:** 1 Basque Centre for Climate Change, Scientific Campus of the University of the Basque Country, Leioa, Spain; 2 Basque Foundation for Science, Ikerbasque, Bilbao, Spain; 3 University of Twente, Enschede, The Netherlands; 4 CEE-M, Institut Agro Montpellier, Univ. Montpellier, CNRS, INRAE, Montpellier, France; 5 CIRAD-UMR G-EAU, University of Montpellier, Montpellier, France

**Keywords:** Environmental sciences, Environmental social sciences

## Abstract

If there is one certainty for the sustainable management of water resources is that facing uncertainty is an unavoidable matter. A concern that, in addition to the best available scientific knowledge and models, requires deep insights about the socio relational processes that underlie decision-making. Our objective here is to better understand if and how the socio relational environment in which decisions are made shapes decision-making under uncertainty in common pool water resource management. Our goal is twofold: methodological and analytical. It consists in designing experiments for carrying out uncertainty analysis to explore the influence that the relationships established among decision actors have in making decision choices under uncertainty in management processes. To this end, we developed one experimental game protocol, representing a typical water management scenario: *irrigation*, which we use to test two different conjectures about the combined effects of uncertainty and relationships. In doing so, we play close attention to the quality of relationships developed among players (acting as water managers), and how these relationships are structured and organized. Initial tests confirmed the importance that the relationships established among players have for coping with uncertainty in managing water resources.

## Introduction

The topic of uncertainty has for a long time been a major concern in decision-making; one that has become particularly relevant in sustainable water management. This has led to the development of a diversity of uncertainty frameworks and conceptualizations across different disciplines and application fields (see^1–8^ for some conspicuous examples). However, despite remarkable advances, making decisions under uncertainty continues to be far from easy. Deep knowledge gaps remain regarding key features of collective behaviors and responses to uncertainty: how decision choices are influenced by the way in which decision-actors relate to one another as well as to the natural system in collective decision-making processes; and how these features can be better characterized and described^9,10^. This raises methodological, conceptual and practical questions about how to better address uncertainty when managing natural resources.

Natural resource management problems have characteristics of wicked problems^11–13^. They exhibit complex dynamics and interdependencies, and are constantly evolving and changing. As such, they are very difficult to model and predict^14^. These problems have no single correct, optimal solution and are often unsolvable^15^. These are problems that are ill-defined, ambiguous and in many cases contested by different actors, rendering different interpretations about which factual knowledge base and uncertainties are valid and relevant, and how these uncertainties must be handled^9^. The case of water management for agriculture, constitutes a good example of a wicked problem, where the supply and demand of water is not independent from farmers’ behaviors, policies, irrigation technologies, market conditions, changes in climate, past decisions; all of which greatly influence how much water is available, who has access to it, and under what conditions this water is accessible.

When dealing with wicked problems, the dynamics of the natural system cannot be considered independent from social behaviors; neither from the formal (e.g., policies) nor the informal rules and values in place (e.g., illegal water extraction), which favor certain actions over others. As several scholars have already suggested^16^, social and natural systems are better conceived as single dynamic socio-ecological systems that are able to adapt and to change; where human actors play the dual role of modifying a system that they are part of, while at the same time changing the rules they create to govern their behavior^17^. In such multi-actor-settings, uncertainties in the natural system are enmeshed with those related with the social system.

Even though these ideas are not new, and have been extensively acknowledged in the scholarly literature (e.g.,^5,18–20^), methodological and practical applications for managing uncertainty are still struggling to take them into account. There remains a tendency to focus on characterizing and modelling uncertainties in the natural system, keeping the social aspects separated. While there is no doubt that doing so can help decision makers better understand the functioning of the biophysical aspects of the managed system, here we will argue that separating these factors generates insufficient conditioning for management^21^. This knowledge cannot explain how the intricate relations forged among social actors and the natural system influence management choices and their outcomes (e.g., agreement of strategies, identification of pressuring issues, setting managing objectives, etc.). Deciding under uncertainty goes beyond making individual rational decisions based on the best available bio-physical knowledge. This is a deeply intuitive, creative, social, political and situational process, that not only depends on the knowledge and preferences of the decision actors, but also on the relationships that connect them. It is through the working of these relationships, played out through the formal and informal institutional arrangements in place, that actors define what is at stake, what must be done and what must be known^22^.

We believe that to better understand the effects of uncertainty in collective decision-making processes, we should analyze uncertainty contextually and take into account the relational complexity of the decision environment in which choices are made. Here, we argue that the quality of relationships established among decision actors, and how these relations are organized, influences how water is managed and the role and function uncertainty plays in it. Working towards this end, we use social experimentation and games to simulate different decision environments, which represent typical managing situations, allowing us to explore the effects of relationships in collective decision-making.

Next, we present basic concepts regarding relationships, uncertainty and resource dilemmas. Building on these ideas we explore three conjectures, about the quality and organization of relationships and their influence in management.

### A background on relationships and uncertainty

#### Relationships

Relationships are what tie people together in a social group. They refer to the links through which people and the surrounding world connect to each other and organize their actions^23^. It is by relating with one another that people make sense of their experience, developing mutually reinforcing interpretations, beliefs, and assumptions about what is going on in particular situations and moments in time; which further manifests in people's behaviors and actions. Relationships are not fixed; they unfold from ongoing social interactions. These interactions are driven by the intentions and understanding of the individuals, as they arise from the tasks in which individuals engage, for example in managing water. In doing so, individuals take on the dual role of carrying out actions and being subject to the actions of others^24,25^. These interactions generally, although not necessarily, materialize through communication.

As such, relationships are shaped by the intersubjective exchange of thoughts and feelings between people (sensu Husserl), constituting also an affective connection and carrying a dimension of emotion and value that reflects back into people’s experiences^26^. Lejano^27^ argues that relationships go beyond what can be discerned in material transactions (e.g., two people exchanging information, goods, resources or actions), including the exchange of cues, feelings, beliefs from which people constitute their identity in connection to one another. Relationships are what bridge the individual with the collective.

#### Uncertainty

Uncertainty is viewed from the perspective of the decision maker, who, being in relationship with (influencing and being influenced by) other actors, has to make decision choices without having a complete and unique understanding of the managed system^5^. For example, a farmer deciding on a cropping plan based on uncertain accounts of water availability and market opportunity; or a water authority setting water quotas for a region without knowing exactly how much water is available and how much it will be used, by whom and where; etc. From this perspective, uncertainty refers to what is not known about the managed system, including (not) knowing what others actors may think and do. It also acknowledges that in multi-actor decision settings ways of knowing are not exclusive, since actor’s interpretations of what is going on and what needs to be done may differ from one actor to another^1,28–30^.

### The study of uncertainty in resource dilemmas

Acknowledging the complexity of socio-technical-natural system interactions, uncertainty has been studied in the context of resource dilemmas, such as common pool resources (e.g., water for irrigation) or public goods (e.g., water infrastructures) (^31–37^ to mention few conspicuous examples). These dilemmas focused mainly on the conflict between self-interest and the welfare of a group when making decision choices, and as such, are central to water management and the point of departure of this work. While our aim is not to conduct a comprehensive review (for this refer to^38^), in Supplementary Review S1, we acknowledge works that have influenced our approach.

This scholarly work confirms that uncertainty and communication are highly significant in natural resource management, clearly showing that the effects they can have, although not always commensurable, can change how people collaborate and interact with each other, reflecting how a system is managed. All in all, this research attests that in management, environmental and social processes are interdependent, and so are the intricate links between what actors know and do. What this research does not yet explain is what happens within a group in which decisions are being made as actors relate to one another and how and why these choices are brought about under conditions of uncertainty. In order to understand the role and function of uncertainty and its influence in making decision choices, these ideas raise the fundamental questions: Does the way in which decision actors relate (the quality of relationships and the organization underlying the development thereof) matter in making decisions under uncertainty?

## Methods

Our goal is explorative, consisting in the application of games and social experimentation to simulate and analyze the development of relationships in collective decision settings like CPR management and their influence on the role and function that uncertainty plays when decisions are made. Our rationale is that through games (simulation tools) we can recreate real world situations^39^ explicitly taking into account emerging relationships among players, allowing us to explore, through experimentation, how players experience uncertainty and how they respond to it in making decisions and choices^40,41^.

Our aim is to investigate if the organization of the social interactions enabling the development of relationships can affect their quality, and if so, how these changes in quality are reflected in how players behave and experience uncertainty. This is operationalized through the adaptation and use of an existing irrigation game protocol^42,43^, which allows us to simulate typical water management situations and explore the following conjectures.

### **Conjecture 1**

*The organization of social interactions and the introduction of communication affect subjects’ relational quality.*

### **Conjecture 2**

*Better relational quality within a group reduces the relevance that subjects attribute to uncertainty in a situation of strategic interactions.*

### **Conjecture 3**

*Better relational quality within a group improves the management of the common pool resources.*

The experimental results are analyzed by carrying out conventional statistical analyses on the game variables, accompanied by a qualitative analysis of the relational quality and relevance of uncertainty as perceived by the players. The qualitative analysis is based on a questionnaire (Supplementary Questionnaire S5) answered by players at different points in time during the game (explained under Treatments and experiments set up). The experiment took place at Montpellier Laboratory for Experimental Economics (LEEM), France. The experimental protocol was approved by the LEEM’s review committee, who ensured that it was in compliance with the rules of experimental economics and the corresponding ethical rules. This committee agreed for the experiments to be carried out within the LEEM platform. The data collection for the experiments complies with the EU General Data Protection Regulation principles; participants signed a consent; data were provided to the research team in total anonymity on subjects.

Next, we present the game protocols, treatments and experimental set-up. Details on the experimental design and practical procedures are added in Supplementary Methods S2 and S4, respectively.

### Game protocol

#### Conceptualization and design

The irrigation game is inspired by an agricultural area that lies along the Têt River, in the region of the Pyrénées Orientales in the south of France, where a small community of irrigators use water from a canal. The main source of water comes from an old canal network, consisting of a primary and a secondary canal. The secondary canal, along which farmer’s plots are located, receives the water inflows from the primary canal. Upstream from it, a reservoir regulates the inflows of water into the network. Water inflows in the area are known for being variable and uncertain, so there is no guarantee of a constant water level in the canal. To avoid water leakages and diminished efficiency, these two networks need to be constantly maintained. For the primary canal, maintenance is covered by water tax contributions from people living in the area. Differently, the maintenance of the secondary canal is the responsibility of the farmers extracting water from it and must be maintained by the farmers themselves.

**Figure 1:**
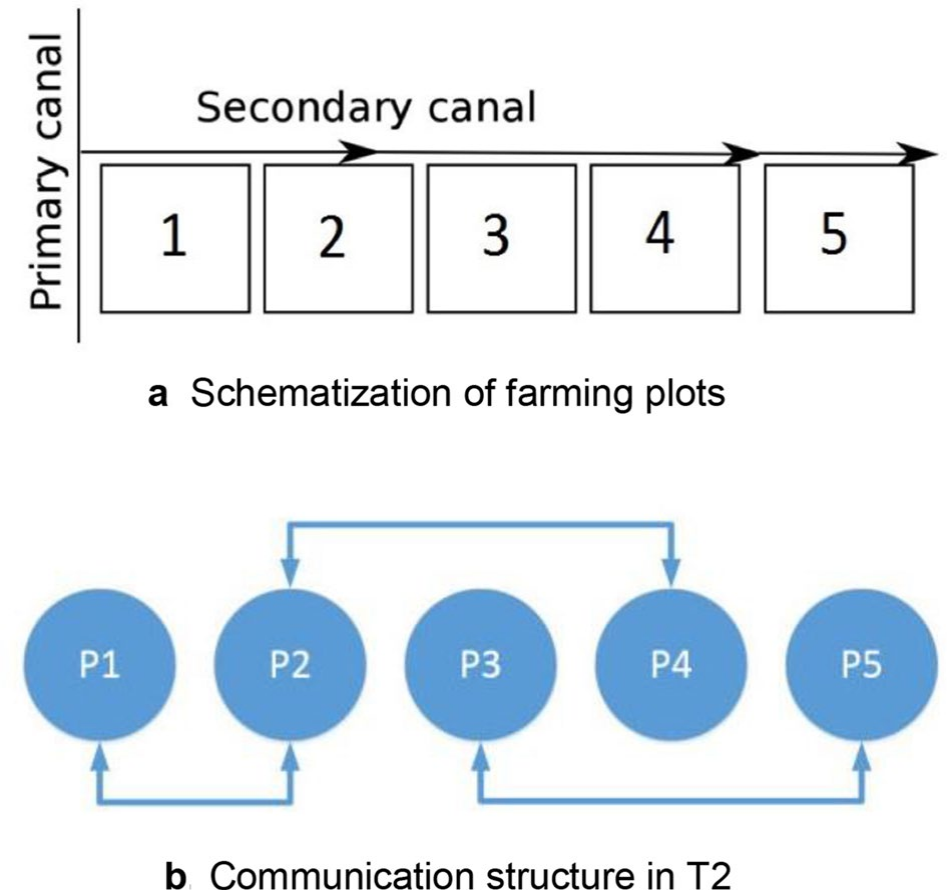
Schematization of farming plots and communication structure in T2.

This game is designed as a combination of a public good and a common pool resource game, based on the experimental design of Janssen et al. and Cardenas et al.^42,43^. It consists of a (secondary) canal, with N farming plots located alongside it (N = 5; Fig. 1a). This canal represents an asymmetric water resource, from which players (farmers occupying the farming plots) extract water for irrigation in sequential order following the player's assigned positions. The player located nearest the canal’s inlet (the most upstream player) is the first one taking water from the canal, followed by the second player, and thus subsequently until the last player downstream is reached. In this sequential scheme, the water available for a player to take is the total water available in the canal minus the water already taken by the upstream players.

Players can extract from zero to the maximum water available at specifically given times. Players only know how much water is available to them individually, but do not know how much water has been taken by the other players or how much is left. Every round, there is an inflow of water at the canal’s inlet, that constitutes the water available for irrigation in total (see Supplementary Fig. S6A). It consists of an average of 100 water units (WU). The input water is unknown to the players. Part of this water input can suffer losses if the canal efficiency is not optimal. The efficiency of the canal is determined by its maintenance, carried through the collection of voluntary individual investments. Efficiency is calculated every round as a function of the total maintenance investments from the previous round. The efficiency is 50% when the total investment is below or equal to 5 ECU (experimental currency unit), linearly increasing up to 100% when investments reach 25 ECU. These values are set as a function of the expected gain of the game, and are known to all players. The maintenance investments must be about 50% of the expected gain in order to optimally maintain the canal. An efficiency reduction results in water leaking from the canal. When the canal has 80% efficiency, 20% of the initial input of the system is lost. This loss is represented by a 20% decrease in the water input received by the first player. Supplementary Fig. S6B, shows the maintenance curve.

One water unit extracted is equivalent to a payoff of one ECU. When players are unsatisfied with the allocation of water, they can stop the game by putting in place a sort of ultimatum, nullifying the gains of the current round and skipping to the next round of the game. When the ultimatum is used, everybody’s gains in the group are set to zero for the round. The public good mechanism of the game is thought to capture the collective actions relating to the maintenance of the secondary canal.

Players do not know each other. Before starting, they are randomly assigned a position alongside the canal that is kept fixed throughout the game. But in all cases they know that a group is always formed of five players. In each round, players must make three decisions: (1) how much water to extract, (2) how much ECU to invest in the canal’s maintenance, and (3) whether or not to use the ultimatum. Players can communicate and exchange information differently depending on the treatment (see subsection “Treatments” below). The game is repeated for 20 rounds, and the scheduled duration of each game is 2 h. Subjects earned an average of 20€.

#### Phases, treatments and experiment set-up

The operationalization of the concepts of uncertainty and relationships for experimentation are detailed in Supplementary Methods S3.

### Experimental phases

Experiments are set up in three phases (P1, P2 and P3), with game conditions supporting the development of relationships along P1 and P3. In P1, the game is played for a maximum of ten rounds (it could be stopped earlier if a player chooses to), then, in P2 participants hold an open dialogue for 10 min, where participants can unrestrictedly discuss the game, possible deals and action strategies. The goal of this phase is to accelerate the creation of new relationships, or strengthen the ones already established during chats in P1. After P2, and with a new configuration of relationships in place, players start P3, consisting in another ten rounds (it could be stopped earlier if a player chooses to). After both P1 and P3, all players respond to a questionnaire (Supplementary Questionnaire S5) to elicit players’ perceptions as developed during P1 or P3 respectively.

### Treatments

Three different treatments are set (Table 1), varying the two control variables: (1) information disclosed to participants (which has a reverse relation to uncertainty), and (2) communication enabled among players: who interact with whom, how and when (structure, configuration, type and timing).

**Table 1:** Communication and information conditions in the three treatments by phase.

	Phase 1	Phase 2	Phase 3
T0	No communication	Dialogue	All to all communication
	Minimum Information		No more information
T1	Unlimited 1 to 1 communication	Dialogue	All to all communication
	Minimum information		Disclosed average gain
T2	Structured 1 to 1 communication	Dialogue	Structure 1 to 1 communication
	Minimum information		Disclosed average gain

#### T0

This is the baseline treatment. In P1 players receive the minimum information needed to play (number of players, own location, water flows, how to extract water from the canal, possibility to use the ultimatum and its penalty, investment in maintenance and calculation of gains). No additional information is disclosed during P2 and P3. During P1 no communication is allowed. During P3, after the dialogue held in P2, chat communication among players is unrestricted (players are allowed to communicate with whom they want).

#### T1

In P1 players receive minimum information. No additional information is disclosed during P2. In P3, the average gain is disclosed in round 5. In P1 players can communicate through one-to-one chats with other players without restrictions. In P3, chat communication is open to all.

#### T2

This treatment is like T1 in terms of information disclosure (average gain in addition to the minimal information in T0); but with a different predefined communication structure, connecting players 1 and 2; players 2 and 4; and, players 3 and 5 (Fig. 1b). This structure ensures that not all information is shared by all players and that only pre-established bilateral agreements are possible.

## Results

### **Conjecture 1**

*The organization of social interactions and the introduction of communication affect subjects’ relational quality.*

The organization of social interactions and the introduction of communication affect subjects’ relational quality.

To test this conjecture, we compared the three treatments in P1. In this phase, information is minimum in all treatments, whilst communication is not allowed in T0, unlimited 1-to-1 in T1 and structured 1-to-1 in T2.

**Figure 2:**
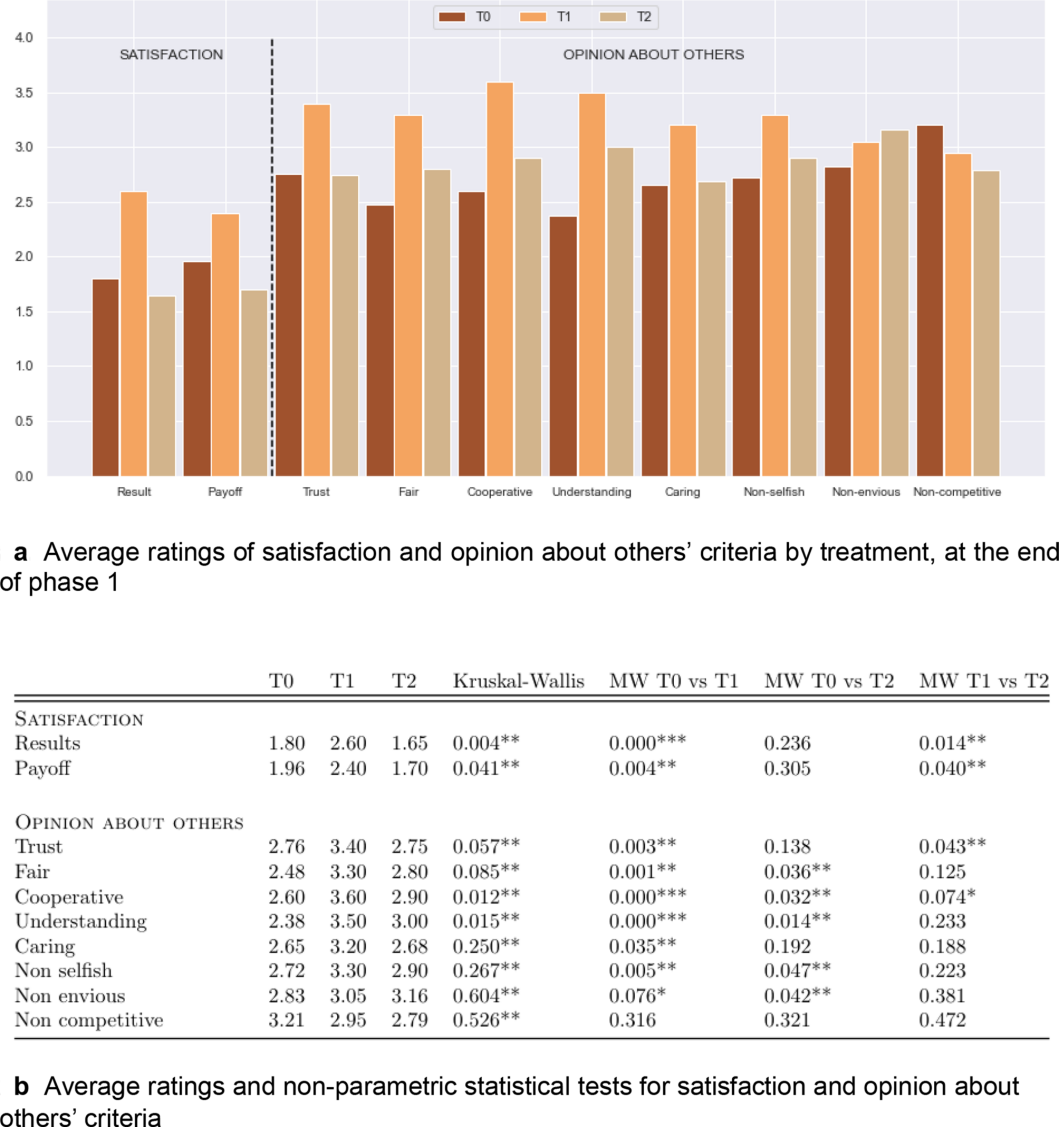
Average ratings of satisfaction and opinion about others’ criteria by treatment, and non-parametric statistical tests.

Figure 2a reports for each treatment the average evaluation of criteria related to *satisfaction,* (player’s level of satisfaction regarding their own performance and gains) and *opinion about others* (players perceptions of others regarding trust, fairness, cooperativeness, understanding, caring, selfishness, enviousness and competitiveness), where for each criterion players faced a five items likert scale. T1 favors both own satisfaction with respect to the game and the positive perception of others on almost all criteria except enviousness and competitiveness. Figure 2b reports the non-parametric tests on the players’ evaluation of each item between the three treatments. The higher evaluation of *satisfaction* in T1 compared to the other two treatments is significant, as well as the perception that other players are trusted and cooperative. The perception that other members of the group were fair, understanding, not envious and not selfish is not significantly different between T1 and T2, but is significantly higher than T0. To complete the analysis, Supplementary Table S7A,B, report the estimations of ordered logit regressions that take into account the position of the player in addition to the communication process. T0 and the first position in the asymmetric game serve as references. The position along the canal does not have much impact on the satisfaction expressed, except for *satisfaction* regarding gains for player 5, the player in the last position on the scheme. When we control for the players' position, T1 also significantly affects the perception of others' cooperativeness and understanding, but not the other variables. Moreover, the position in the game matters: the player at the second position has very positive evaluations of trust, fairness, cooperativeness, caring and non-selfishness about the other players in his/her group. But we can easily imagine that this evaluation mainly concerns his/her opinion about the upstream player. Overall, the 1-to-1 unlimited communication fosters trust in the group for four out of five players.

In summary, the observations are consistent with the conjecture in that allowing interactions via communication within a group helps members improve relational quality. The results confirm an effect of social interactions on player’s *satisfaction* and *opinion about others,* endorsing the idea that the possibilities players have of interacting affect the relationships developed within the group. However, it does matter how these interactions are organized: relationships are better when there is no particular structure imposed, like in unlimited 1-to-1 communication in T1, giving players more freedom in deciding with whom to communicate with.

### **Conjecture 2**

*Better relational quality within a group reduces the relevance that subjects attribute to uncertainty in a situation of strategic interactions.*

Better relational quality within a group reduces the relevance that subjects attribute to uncertainty in a situation of strategic interactions.

To test this conjecture, we examined the questionnaire (Supplementary Questionnaire S5) answers. The results show that changes in relational quality are paralleled with changes in how relevant uncertainty is perceived during the experiments (Supplementary Table S7C). Regardless of the treatment, improvements in the quality of relationships are always accompanied by a decrease in importance players attributed to uncertainty. Uncertainty is regarded as more relevant in the treatments with less connecting possibilities, i.e.: T0 P1 (no communication), and T2 (structured 1-to-1 communication), and, vice versa, it is less relevant in treatments with more connecting possibilities, i.e.: T1 (unlimited 1-to-1 communication). The parallelism between relational quality and relevance of uncertainty is also reflected in the correlation coefficients among these variables, with *communication exchange* and *opinion about others* being positively correlated among each other (correlation coefficient 0.97); and negatively correlated with the relevance of *uncertainty* (correlation coefficients − 0.95 and 0.88 respectively).

### Between analysis

**Figure 3:**
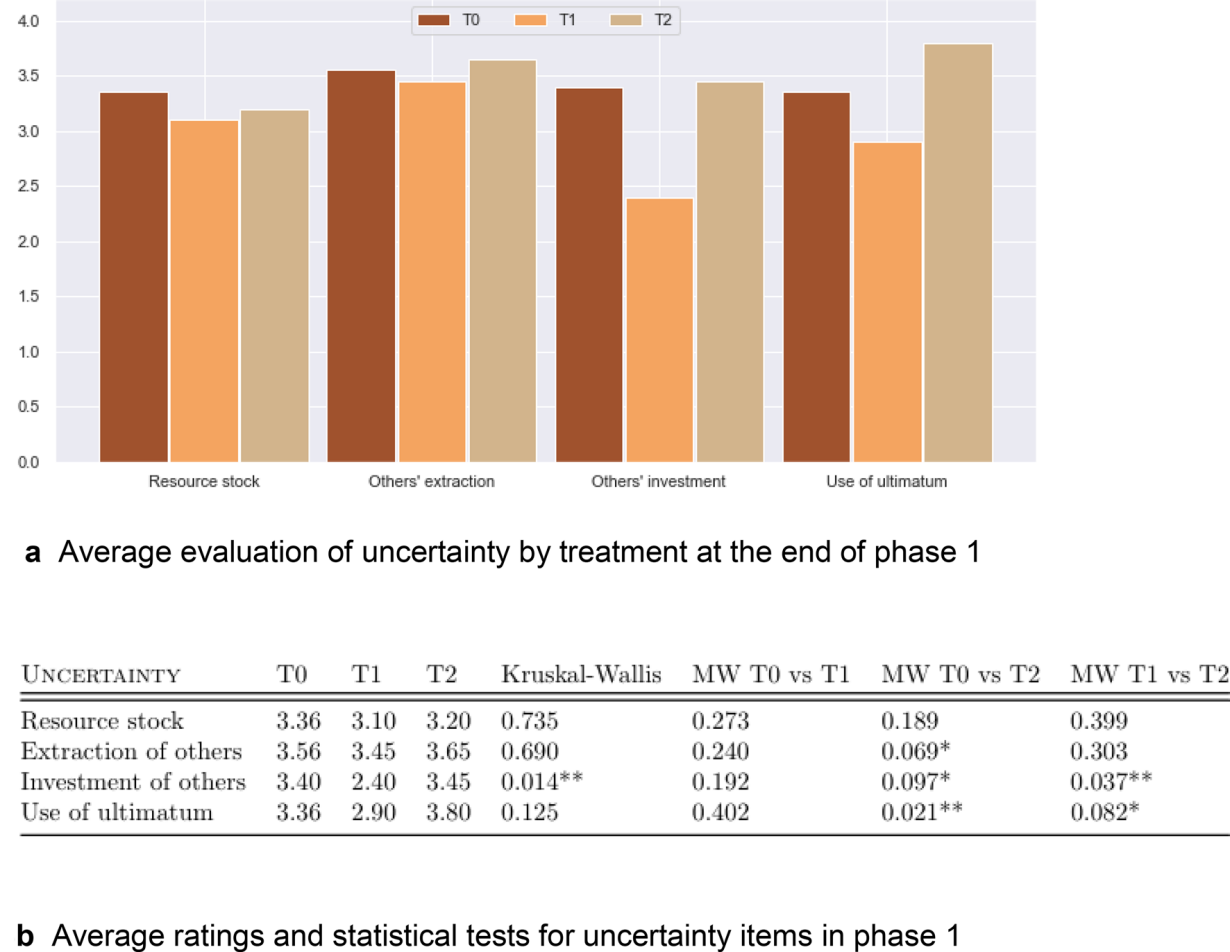
Average evaluation of uncertainty by treatment in phase 1 and statistical tests.

Comparing T0, T1 and T2 in P1, where treatments are subject to the same level of uncertainty (minimum information to play the game and no additional information disclosure), uncertainty is perceived as less important in T1 than in T0 or T2. Figure 3 reports the average evaluations (a) and the non-parametric statistical tests (b) of the four uncertainty criteria: the stock of resource, the extraction chosen by the others, the investment chosen by the others, and the decision to use the ultimatum, stopping the round and setting the payoff of each player in the group to zero. Regardless of the criterion, the mean score in T1, is lower than in the other two treatments. Conversely, T2 appears to foster the perception of uncertainty about the investment decision and the decision to skip the current round sanctioning all group members with zero gains.

### Within analysis

The biggest differences between P1 and P3 are observed under all-to-all communication. T1 is the treatment where the lowest average values of relevance of uncertainty are observed in both P1 and P3. Differently, T0 is where variations in the perception of relevance of uncertainty have the biggest differences between P1 and P3 (Supplementary Table S7C). T0 transitions from no communication in P1 to all-to-all communication, suggesting positive effects of enabling communication even when it was not previously practiced. Looking at the individual uncertainty criteria (Supplementary Table S7D), it is observed that there is a general decrease of relevance of uncertainty in all four criteria between P1 and P3, except in both T1 and T2, where uncertainty about other’s investments becomes more relevant in P3.

### Uncertainty perceived as a function of opinion about others

**Figure 4:**
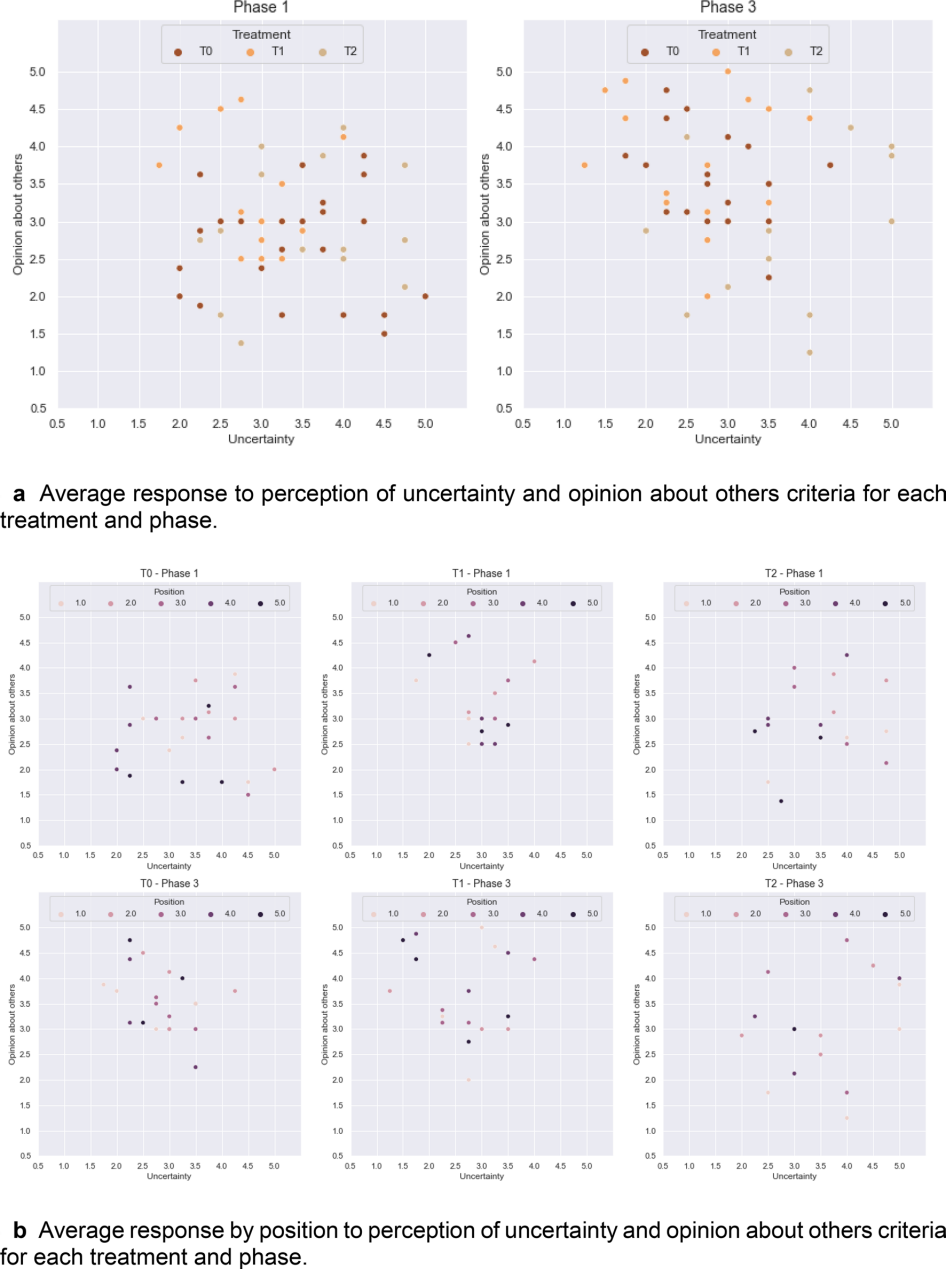
Average response to perception of uncertainty and opinion about others.

**Figure 5:**
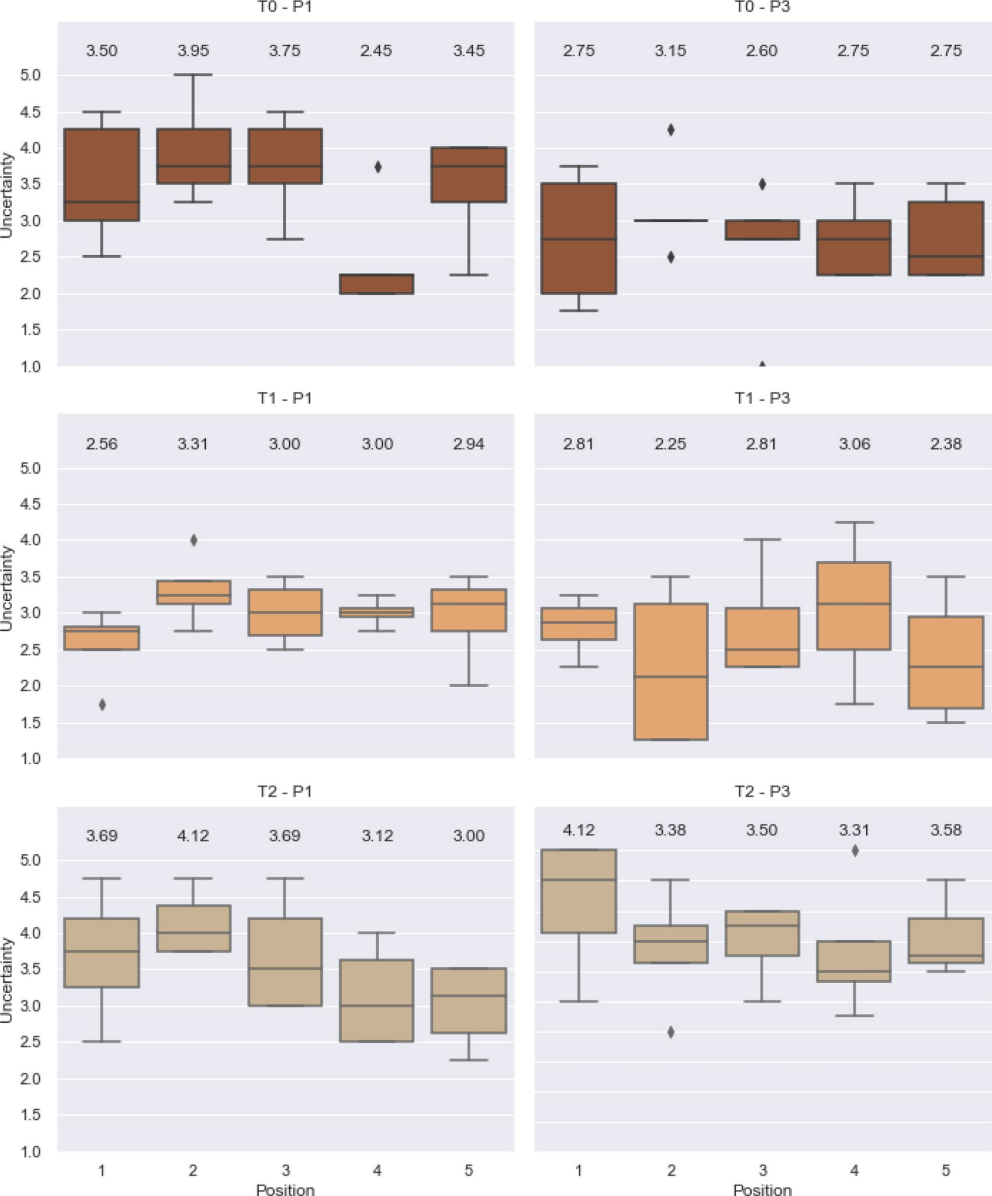
Perception of uncertainty by position (averages above the boxes).

Generally speaking, T1 shows higher *opinion of others* and lower relevance of *uncertainty* improving from P1 to P3, while T2 presents a less desirable pattern, with lower *opinion about others* and high relevance of *uncertainty* (Fig. 4a). The average response by position to the perception of *uncertainty* and *opinion about others* (Fig. 4b) shows clearly differentiating patterns among the three treatments, where T1P1 shows less dispersion of values, high *opinion about others* for all positions and attributes middle range importance to uncertainty. In P3, there is a shift towards a higher *opinion about others* and a lower relevance assigned to uncertainty, led by tail-enders. This is in agreement with the increase in investments from player 5 (conjecture 3). T0P1 is more dispersed, holding lower *opinions about others* than in T1, with the relevance of *uncertainty* reaching its maximum for some head-enders. In P3, where all-to-all communication is allowed, a positive turn is observed, with a higher *opinion about others* and a reduced relevance of *uncertainty*, again being remarkable for position 5 (tail-ender) (Fig. 5). The patterns observed in T2 are yet different. P1 and P3 are similar in their perception of *uncertainty*, remaining middle high and broadly spread in the *opinion about others*; which seem to reflect the effects of the structure underlying players' interactions in T2.

### Reactions to the disclosure of average gain

Changes in the perception of uncertainty were also reflected in how players responded to the disclosure of *average gain*, reducing social uncertainty regarding how much others were gaining; leading to stronger reactions in T2 than in T1, and being different for head-enders than tail-enders. See Supplementary Answers S8 for detailed answers.

Our conclusion on Conjecture 2 is mixed. Indeed, we expected that T1 (unlimited 1-to-1 and all-to-all communication), where relational quality is highest, would significantly reduce the uncertainty for all four criteria. Instead, the relevance of uncertainty seemed to change based on uncertainty type and relational situation (player’s position, player’s capacity to communicate, history of interactions in previous phases). Uncertainty about others' investments increases significantly in T1 and T2 and decreases in T0. Uncertainty about the use of ultimatum decreases between P1 and P3 in all treatments. While uncertainty about other’s extractions became relevant in phase 3, e.g., in T0. Furthermore, reactions to disclosure to *Average gain* were also dependent on players’ position and treatments, with T2 responding with an increased use of the ultimatum, and T1 showing preference for negotiation.

#### **Conjecture 3**

*Better relational quality within a group improves the management of the common pool resources.*

Better relational quality within a group improves the management of the common pool resources.

**Figure 6:**
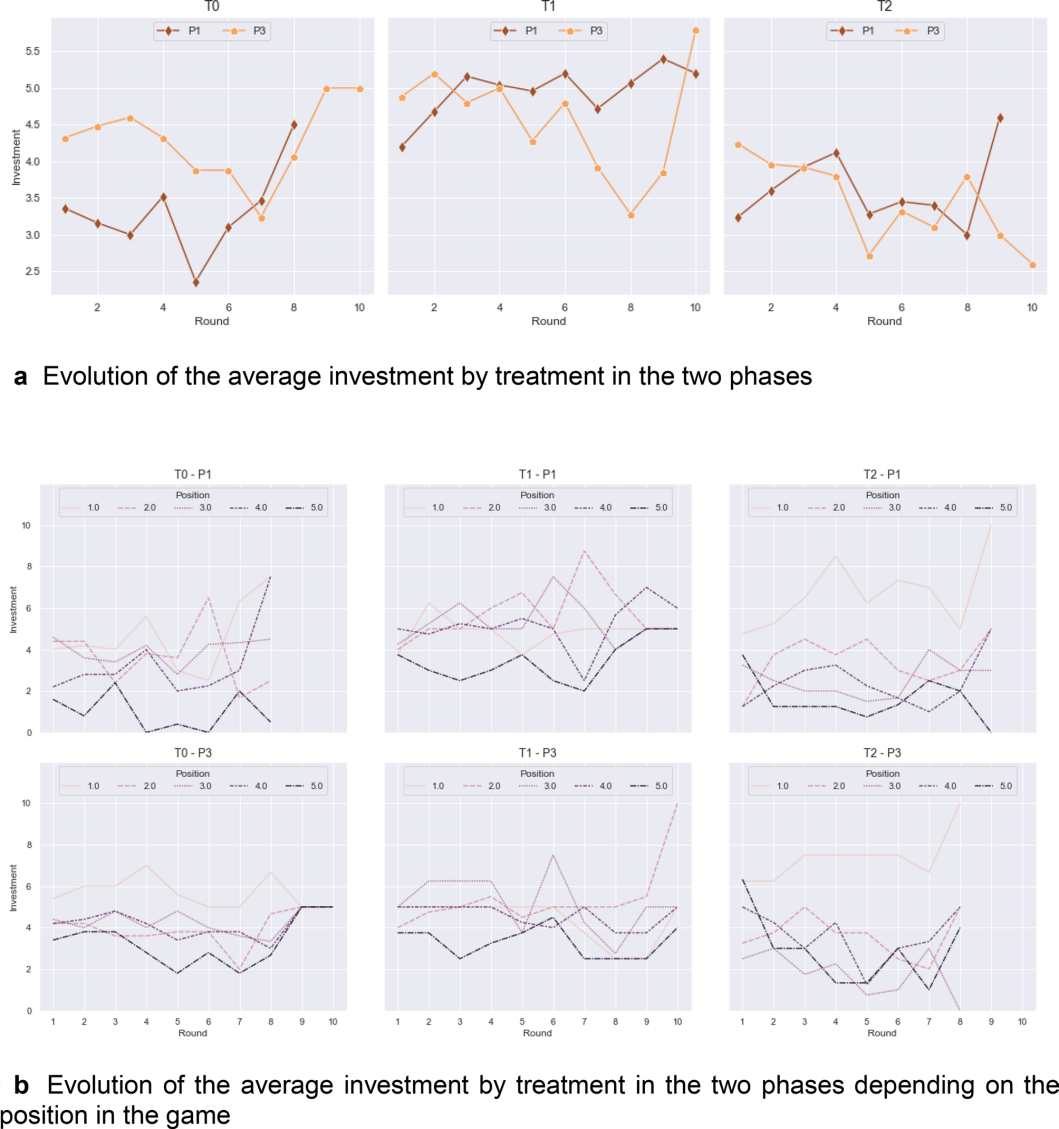
Evolution of the average investment in the two phases by treatment and depending on position.

To test this conjecture, we looked at the average investment, which determines the efficiency of the canal for the next round in the three treatments as it evolved from P1 and P3. The average investment is significantly higher in T1 (unlimited 1-to-1 communication) compared to T0 (no communication) and T2 (structured 1-to-1 communication) (MW T1 vs T0 p-value = 0.016, T1 vs T2 p-value = 0.002), while the latter two are close to each other (MW p-value = 0.273; Fig. 6a). However, it is worth noting that the slope in the three first rounds of treatment T2 and T1 increases, whereas in T0 the slope decreases. Figure 6b provides more details about the investment depending on the position along the canal. We can clearly observe that the player in the fifth position stands out in treatment T1 compared to the other two treatments, with a significantly higher investment (MW T1 vs T0 p-value = 0.017 and T1 vs T2 p-value = 0.009), meaning he/she felt more implicated in the water management.

**Figure 7:**
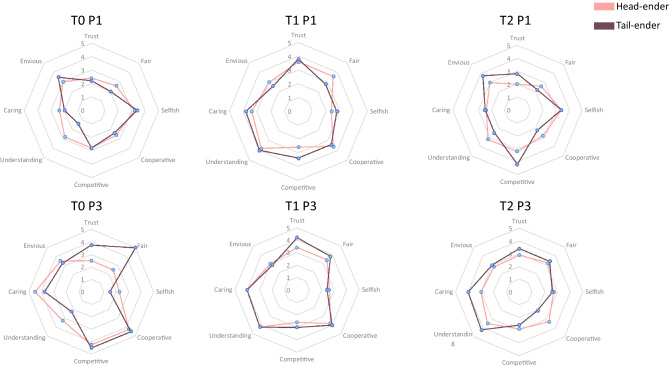
Opinion about others for head and tail enders, per treatment and phase.

A within treatment comparison of the average investment between P1 and P3 (Wilxon test T0 p-value = 0.010, T1 p-value = 0.275, T2 p-value = 0.515) shows a significant increase in T0. In T1 and T2, where a communication structure already existed in P1, this improvement is not observed. This suggests that the introduction of communication in T0 leads to a significant increase in players’s investment to ensure the effectiveness of the canal. In particular in the case of player 5, whose average investment in P1 (0.97) significantly increases in P3 (3.07) (Wilcoxon p-value < 0.001), while at the same time considerably improving the perception of others, increasing in fairness, trust, cooperativeness, caring, competitiveness and investment, and decreasing in envy and selfishness (Fig. 7).

Our conclusion for Conjecture 3 is that all-to-all communication, and therefore better relational quality (Conjecture 1), induces cooperation within a group and better involves tail-enders, improving investments and canal effectiveness.

## Discussion and conclusions

Aiming at a more comprehensive understanding of the role and function of collective decision-making, we questioned the influence of relationships in coalescing decision choices. To this end, we embarked in a methodological and analytical exploration, and trialled social experimentation as a means of investigating synergies between relationships and knowledge in CPR management. Following this explorative goal, and based on already existing experimental designs (i.e.,^42,43^), we developed a game protocol, which we used to set pilot experiments to gather preliminary evidence on the combined effects of uncertainty and the working of relationships. In this work we focused on water, however, our results could be easily extended to other natural resources.

Our findings confirm that social interactions induce a change in the quality of relationships developed among subjects in a group. Overall endorsing the proposition that the more possibilities of interaction the better the relational quality that unfolds. A proposition that beyond being general, is restricted by how social interactions are organized. Our results show that less controlled interactions induce better relational quality. Particularly concerning players’ *satisfaction* and *opinion of others*. In our experiments players developed better relationships when given the possibility to self-organize with whom to communicate, rather than when these interactions were predetermined, e.g., structured 1-to-1 communication. In phase 1, the groups in T1 developed more trust and cooperativeness and were more satisfied with their own performance and gains than in those in T0 and T2. We also observed that an additional change occurred when communication was fully out ruled in T0, where the gathered *opinion of others* was significantly less fair, less understanding, more envious and more selfish than in T1 and T2.

Changes in the quality of the relational environment were accompanied with changes in how uncertainties (about: resource stock, others extractions, others investments, use of the ultimatum) were perceived, with lowest mean scores in T1, the treatment with most communication. The relevance players assigned to each uncertainty varied depending on the treatment and phase. Uncertainty about investments being lowest in the treatment with the highest relational quality and the most even distribution of investment among players (T1). This suggests that the development of relationships goes hand in hand with higher levels of trust and cooperation in contributing to the greater community. Differently, uncertainty about the ultimatum use is relevant in T2, the treatment that shows the least even distribution of investment, extraction, and earning, and additionally where players used the ultimatum most. Furthermore, the answers from the questionnaire show qualitatively different responses to the disclosure of *average gain*, reinforcing the notion that good quality relationships, like in T1, can lead to responses that are qualitatively different, supporting dialogue and negotiation and less use of ultimatum.

Our results also reveal that improving relational quality is not just a matter of letting people freely communicate. The communication exchange builds on previously formed relationships, and is shaped by past interactions. This is for example what is observed in T0 and T1, where both treatments have all-to-all communication in P3, but differ in P1 (no communication in T0 and unlimited 1-to-1 in T1). While the performance as a group is not significantly different, there is bigger improvement in efficiency, reduced use of ultimatum and investment in T1 than in T0.

Looking at players individually, points out tail-enders behaving differently than the other players. Earning significantly less and having less available water in P1, tail-enders are considerably less satisfied. However, as they are able to interact with others more, they become equally satisfied with the other players in P3. This is paralleled by an individual and collective increase in investments and gains as well as a decrease in ultimatum use. Tail-enders improve their *opinion about others*, becoming more cooperative, less preoccupied with uncertainty, more understanding of the other players and investing more. Arguably good relationships provide an organic structure for the regulation of asymmetries, giving players at the tail-end the chance of being more equally integrated, incrementing the average players’ investments. We also observed that players preferred to negotiate strategies rather than to voice their opinion through the use of ultimatums; with the downside of increasing tolerance to freeriding.

The positive association between players’ interactions and an increased relational quality, must be interpreted in the context of the game, where there is no history of previous collaboration among players, a factor which in real life could greatly influence the development of relationships and following actions. Furthermore, it could also be argued that players’ behaviors and the development of cooperative strategies through the game are a mere result of learning effects. Against this argument though, we observed that player’s interactions were always accompanied by a change in the appreciation they have for each other, which beyond learning how to play the game, denoted a human capacity to recognize themselves and others in the pursuit of a common endeavor and inclusivity.

The novelty of this work does not reside in using social experimentation to investigate decision making under uncertainty per se, a topic that, as indicated in the sections above, has already been extensively investigated in CPR, but in explicitly considering relationships as a constitutive element in collective decision-making processes. Where relationships, beyond serving the purpose of exchanging information, connect people, their experiences and feelings, eventually influencing player’s behaviors. In doing so, bringing a new dimension into the understanding of uncertainty, one that changes the focus of analysis from individual to group level dynamics. Relationships represent connections among players, formed through organized interactions that enable them to communicate. Moreover, serving to exchange information, they constitute a qualitative person-to-person connection that reflects the player’s lived experience as they interact with one another.

From this experience, we conclude that social experimentation and gaming constitute a promising venue for studying the effects of uncertainty and social relational processes in decision-making settings. While these experiments served our exploratory purpose, future studies must increase the number of observations in order to ensure stronger inferences, as controlled experiments show potential in analyzing new research questions. Our results also showed that the role and function of uncertainty is context and time dependent, with different uncertainty types becoming relevant in different situations, therefore we suggest prospects analyses carefully differentiate among uncertainty types and when they occur. Overall, this methodology provided us with a comprehensive analytical framework where the existing interdependencies among people, including nature and technology could be explicitly modelled, allowing exploration of different configurations and structuring of relationships. Furthermore, including real people to participate in the experiments made it possible to account for tacit and emotional aspects of knowing, and to therefore determine the quality of the relationships in which players engaged.

These outcomes also provide new insights for CPR management. In our experiments enabling the development of relationships improved the way players viewed themselves in connection to others, and associated with more sustainable decisions (e.g., more investment in maintenance). This suggests that addressing uncertainty in collective decision-making processes is not just a matter of gathering more and better information about the managed system, but it also requires tending to how relationships among actors are configured and organized in defining what is at stake and what must be done. In our results, high quality relationships were always associated with the best game outcomes (more sustainable and equitable management, better distributed resources, less ultimatums used), suggesting that investing in the development of good relationships (e.g. dialogue and negotiations) as a complement to already existing strategies of data collection can be a good practice for finding viable solutions to common pool problems. For answering our initial driver question: Does the way in which decision actors relate (the quality of these relationships and the organization underlying the development thereof) matter in making decisions under uncertainty? Our gathered preliminary evidence through this pilot case, so far suggests: Yes, it does.

## Supplementary Information

Supplementary Information.
