# Supplementary material for: Relational quality and uncertainty in common pool water management: an exploratory lab experiment
Source: Sci Rep. 2021 Jul 26;11:15188. doi: 10.1038/s41598-021-94517-6 (PMC8313544; doi:10.1038/s41598-021-94517-6)
Supplement: Supplementary file 1 — Supplementary Information. [file 41598_2021_94517_MOESM1_ESM.pdf]

Supplementary Information for

Relational Quality and Uncertainty in Irrigation Water Management: an exploratory lab experiment

Marcela Brugnach, Sander de Waard, Dimitri Dubois, Stefano Farolfi

This PDF file includes:
Supplements S1 to S8

Supplementary Review S1. The study of uncertainty in resource dilemmas
Supplementary Methods S2. Exploring designs
Supplementary Methods S3. Operationalization of the concepts of uncertainty and relationships
Supplementary Methods S4. Practical procedure
Supplementary Questionnaire S5. Questionnaire and qualitative variables
Supplementary Figures S6. Figures
Supplementary Tables S7. Tables
Supplementary Answers S8. Answers to average gain disclosure

Supplementary Information for

Relational Quality and Uncertainty in Irrigation Water Management: an exploratory lab experiment

Supplementary S1. The study of uncertainty in resource dilemmas

Biel and Garling (1995) (1), investigated the role uncertainty plays in whether people defect or cooperate. They argue that the effect of environmental uncertainty (uncertainty associated with the natural system) is not independent from the value orientation of the individuals that are affected by it. While individuals with pro-social values act in the interest of the collective and cooperate, others only do so if the situation imposes limitations in their egoistic or individualistic behavior, through social pressure, communication, rewards or penalties. So, for an egoistic person, increasing uncertainty may increase defection (individual acting in self-interest), making cooperation less consistent. Following similar lines of thought, Gustafson et al (2000), Rapoport et al. (1992) (2, 3), also found out that in public good games uncertainty reduced the level of cooperation. According to this research, how people respond to unknowns in the natural system is influenced by social uncertainties (e.g., not knowing what others do) affecting their ability to cooperate and act in concert.

Expanding these studies of the effects of uncertainty to asymmetric access resources, such as upstream-downstream irrigation systems, Anderies et al 2013 found out that when access to resources is not symmetric, uncertainty reduces cooperation. They investigated how micro-situational variables (uncertainty and heterogeneity) affect cooperation, hypothesizing that uncertainty may induce head-enders to be more cooperative and exploring how group composition could affect their capacity to solve a problem. Their results show that variability leads to low level earnings and declined performances, and suggest that uncertainty can amplify fragilities, with potential large shocks inducing non-cooperative behaviour that becomes catastrophic. With these results Anderies et al (2013) (4), as well as Farolfi et al. (2014) (5) uncovered the importance of context in decision-making.

Another critical factor in the self-governance of shared resources under conditions of uncertainty, and one that received extensive attention, is communication (6-8). While correlated positively with cooperation and the reduction of overharvesting of common resources (6, 9), the effects of communication have also been reported as being greatly influenced by how much we know about what others do and the state of the resource being managed. Focusing on the role of information and communication in governing common pool resources, Janssen (2013) (10) explored these issues, investigating the effects of limited information regarding the actions of others (i.e., social uncertainty) and resource availability (i.e., environmental uncertainty) in collective actions in a socio-ecological system. He reported that limited information affects cooperation and lowers the level of compliance, suggesting that participants create institutional arrangements that fit the level of visibility they experience. In his analysis he considers communication as a fundamental factor for coordinating and aligning expectations among actors' actions. He argues that communication can change the effects of limited information, since the lack of monitoring of other's actions can reduce the level of compliance with informal institutional arrangements. Similar results are shown in Janssen et al. (2015) (8), where focusing exclusively on irrigation dilemmas, they found that constrained communication can cause inequalities, and an eventual lack of ability of a group to generate a shared problem comprehension. Finally, it is worthwhile mentioning the findings by Dubois et al. (2020) (11) who identify contrasting, not always positive, effects of sharing information in CPR management.

Following this rationale, in a meta-analysis on the governance of common resources, Janssen (2015) (8) refers to the work of Fischbacher et al. (2001), Janssen et al. (2014), Fehr and Camerer (2007) (12-14), claiming that collective actions analyses must be made based on assumptions about individuals holding imperfect knowledge, with the capacity to adapt and change, and be influenced by their

contexts. He suggests that recognizing the influence of the assumptions and expectations people hold about others when cooperating (other-regarding preferences and conditional cooperation) is essential in acknowledging that, while there is a tendency for people to cooperate when others also do so, people also cooperate because it is valued and emotionally beneficial. He emphasizes the importance of communication -cheap talk- and its effects in enabling participants to signal their intentions and trustworthiness.

References

1. Biel, A. & Gärling, T. The role of uncertainty in resource dilemmas. *J. Environ. Psychol.* **15(3)**, 221-233 (1995).
2. Guftansson, M., Biel, A. & Gärling, T. Overharvesting of resources of unknown size. *Acta Psychol.* **103(1-2)**, 47-64 (1999).
3. Rapoport, A., & Au, W.T. Bonus and Penalty in Common Pool Resource Dilemmas under Uncertainty. *Organ. Behav. Hum. Decis. Process.* **85(1)**, 135-165 (2001).
4. Anderies, J. M., Janssen, M. A., Lee, A. & Wasserman, H. Environmental variability and collective action: Experimental Insights from an irrigation game. *Ecol. Econ.* **93**, 166-176 (2013).
5. Farolfi, S., Désolé, M., & Rio, P. Influence of Context on Player Behaviour: Experimental assessment. *Simul. Gaming* **45(4-5)**, 627-665 (2014).
6. Balliet, D. Communication and Cooperation in Social Dilemmas: A Meta-Analytic Review. *J Conflict Resolut* **54**, 39-57 (2010).
7. Ostrom, E. Understanding Institutional Diversity. *P.U.P.*, Princeton, New Jersey, USA (2005).
8. Janssen, M. A., Anderies, J., Perez, I., Yu, D. J. The effect of information in a behavioral irrigation experiment. *Water Resour. Econ.* **12**, 14-26 (2015).
9. Poteete, A. R., Janssen, M. A. & Ostrom E. Working Together: Collective Action, the Commons, and Multiple Methods in Practice. *P.U.P.*, Princeton, New Jersey, USA (2010).
10. Janssen, M. A. The Role of Information in Governing the Commons: Experimental Results. *Ecol. and Soc.* **18(4)** (2013).
11. Dubois, D., Farolfi, S., Nguyen-Van, P. & Rouchier, J. Contrasting effects of information sharing on common-pool resource extraction behavior: Experimental findings. *PLOS ONE* **15(10)**, e0240212. https://doi.org/10.1371/journal.pone.0240212 (2020).
12. Fischbacher, U., Gächter, S. & Fehr, E. Are People Conditionally Cooperative? Evidence from a Public Goods Experiment. *Econ. Lett.* **71(3)**, 387-404 (2001).
13. Janssen, M. A., Tyson, M. & Lee, A. The effect of constrained communication and limited information in governing a common resource. *I.J.C.* **8(2)**, 617-635 (2014).
14. Fehr, E. & Camerer, C.F. Social neuroeconomics: The neural circuitry of social preferences. *Trends Cogn. Sci.* **11**, 419-427 (2007)

Supplementary Information for

Relational Quality and Uncertainty in Irrigation Water Management: an exploratory lab experiment

Supplementary S2. Exploring designs

According to Barreteau et al. (2007) (1), a game must be designed as a specific instance of a conceptual model, representing the socioecological system and the resource at stake. Furthermore, it needs to be designed in such a way that it reflects the dynamics of the real system, allowing complex interactions and communication among players. For our specific purpose, a game must offer the possibility of setting experiments that allow to explore if and how the relational environment developed through player´s engagement with one another influences the decisions they make under conditions of uncertainty. Designing such a game implies to explicitly account for ways of letting relationships among players to unfold, and of keeping track of how these relationships influence player´s experiences and practices.

This led us to consider the organizing processes of interactions underlying the development of relationships and the quality of relationships, as two essential aspects of our experiments. Organization, referring to how players are able to connect with each other; including structure (e.g., who interacts with who?), configuration (e.g., open communication, in sequence), type (e.g., communication through written messages, face to face), timing (when players connect with each other). Quality, referring to how players value their experience in playing the game, regarding their level of satisfaction in how they play, their perception of uncertainty, and how they communicate with each other, and what they think and feel of themselves and the others as they engage in playing.

Our design work started with an interdisciplinary workshop, held at the former French National Research Institute of Science and Technology for Environment and Agriculture (IRSTEA, now INRAe) in January 2015, where circa 50 researchers of this institution with varied expertise (modellers, biologists, economists, sociologists, agronomists) participated, with the aim of identifying the specific cases, that, being of common interest to these researchers, could be used as a subject matter for our games. From the various options suggested, one case was selected for further development and follow up: An irrigation game, which in what follows, we use as the pilot case for experimentation. In these complex problem settings, uncertainties occur at many different levels: relating to the individual actors, the social system or network, the context, the environment, the institutions, the economy, the natural environment, the options, solutions, technologies, reality versus representations or information, current / future / past narratives. As part of these collaborative efforts, we also identified the uncertainties to be considered.

Reference

1. Barreteau, O., Le Page, C., & Perez, P. An introduction: Contribution of simulation and gaming to natural resource management. *Simul. Gaming* **38**, 185 (2007).

Supplementary Information for

Relational Quality and Uncertainty in Irrigation Water Management: an exploratory lab experiment

Supplementary S3. Operationalization of the concepts of uncertainty and relationships

Operationalization of uncertainty: Uncertainty stands for what players do not know regarding the management of the CPR system, concerning the water resource, the canal, and others´ behaviours. So, uncertainty refers to lack of knowledge about what others are doing (e.g., water extractions, gains and investments, or use of ultimatum), the state of the water system (e.g., water available for extraction) and the technical system (e.g., canal´s level of maintenance). Each treatment varies in what information is disclosed to participants and when, as indicated below. *Average gain* is disclosed in T1 and T2 during Phase 3, reducing the uncertainty regarding how much others are gaining and how much water they are using.

Furthermore, it is important to mention that even though these are designed as deterministic (non-stochastic) games, players at time *t* do not know, for example, how much water there will be at time t+1. For example, a period of drought is simulated, by lowering the input of water to the canal during the last phase of the game, which players do not know about (see Supplementary Figure S6 A).

Operationalization of relationships: The development of relationships in the experiments is made possible through organized interactions carried out via communication among players. In the experiments interactions are configured, structured and timed differently through the treatments (unlimited or structured 1- to -1, all-to-all communication, carried out via online chat), as indicated in the design of the treatments per game in the section below.

The quality of relationships is established through elicitation of the following qualitative variables: 1. player´s level of satisfaction regarding their own performance and gains; 2. their perception of others in connection to themselves and. of themselves in connection to others (based on attributes, such as: trust, caring, understanding, fairness, competitiveness, cooperativeness, enviousness and selfishness), 3.their experience communicating with others, and 4. the relevance players assign to uncertainty, regarding initial water inputs, how much water others extract, how much they invest in the canal and ultimatum use. This information is elicited via a questionnaire (Supplementary Questionnaire S5) rating answers in a 5 items likert scale. It is assumed that players do not know each other before the game, so there is no previous relationship formed among them, or past common history of collaboration.

Supplementary Information for

Relational Quality and Uncertainty in Irrigation Water Management: an exploratory lab experiment

Supplementary S4. Practical procedure

A total of 75 subjects (53% women) participated in the experiment. Participants were students from University of Montpellier, randomly selected from a pool of volunteers[1], from various educational backgrounds and without previous experience. The experiment was computerized and took place in the experimental economics laboratory of Montpellier (LEEM), which is composed of 20 terminals isolated from each other by boxes. Players could communicate only through the computer no visual contact or direct communication was possible. The experimental session lasted for 2 hours including instructions and payment. A total of 3 experimental sessions were held. The game was played by 15 groups of 5 subjects each, with 5 groups allocated to each of the 3 treatments. Subjects were allocated to treatments following a ‘between design’ (different subjects for each treatment). We are aware that 5 groups per treatment falls below the standard of 8 or 10 groups per treatment used as a rule of thumb in lab experiments, but we consider that for our explorative purpose, a broader spectrum of treatments rather than groups/individuals per treatment, was more suitable to identify trends and compare different situations. We expected that the combined effects of relationships and knowledge, we are looking to identify, will be apparent in even small samples.

---

[1] The database of volunteer participants is managed with the ORSEE platform (Online Recruitment System for Economic Experiments - Greiner 2004, 2015).

Supplementary Information for

Relational Quality and Uncertainty in Irrigation Water Management: an exploratory lab experiment

Supplementary S5. Questionnaire and qualitative variables

Questionnaire

Satisfaction
Where you satisfied with your performance during the game?
Where you satisfied with the amount of profit you gained during the game?

Uncertainty
How important was (not) knowing the initial water input to you?
How important was (not) knowing the abstractions of others to you?
How important was (not) knowing the investments of others to you?
How important was the threat of others stopping the round to you?
Did the disclosure of the average gain change your opinion of other players? If so, how?

Ultimatum use
If you pressed stop: Why did you do so?

Phase 2
Did the dialogue with other players (phase 2) influence your opinion of these players? If so, how?

Communication exchange
How was the communication with other players?
Difficult – Easy
Confusing – Clear
Not useful – Useful

Opinion about others
How would you describe the behavior of other players towards you?
Not trustworthy – Trustworthy
Not Fair – Fair
Selfish – Not selfish
Not cooperative – Cooperative
Competitive – Not competitive
Ignorant – Understanding
Neglecting – Caring
Envious – Not envious

Opinion about themselves
How would you describe your behavior towards the other players?
Not trustworthy – Trustworthy
Not Fair – Fair
Selfish – Not selfish
Not cooperative – Cooperative
Competitive – Not competitive
Ignorant – Understanding
Neglecting – Caring
Envious – Not envious

Qualitative variables

Satisfaction: level of player's satisfaction regarding their own performance and gains.

Relevance of uncertainty: importance players attribute to uncertainty.

Communication exchange: how players experience the communication with other players, as an average considering how easy, clear and useful.

Opinion about others: opinion players construct about the other players to whom they relate, computed as an average of different relational qualities: trustworthy, fair, selfish, cooperative, competitive, understanding, caring, envious

Opinion about themselves: formed opinion regarding themselves in relation to others, computed also as an average of the qualities: trustworthy, fair, selfish, cooperative, competitive, understanding, caring, envious.

Answers were rated in a scale from 1 (very bad) to 5 (very good).

Supplementary Information for

Relational Quality and Uncertainty in Irrigation Water Management: an exploratory lab experiment

Supplementary S6. Figures

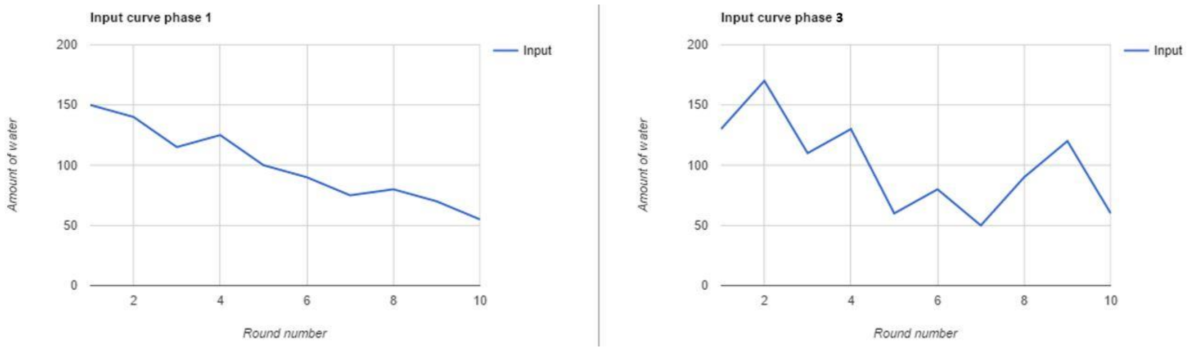

Figure A. Water input curve for phases 1 and 3. In phase 1 water input reduces over 10 rounds, simulating a situation of water scarcity. In phase 2 water input is variable but with a constant average

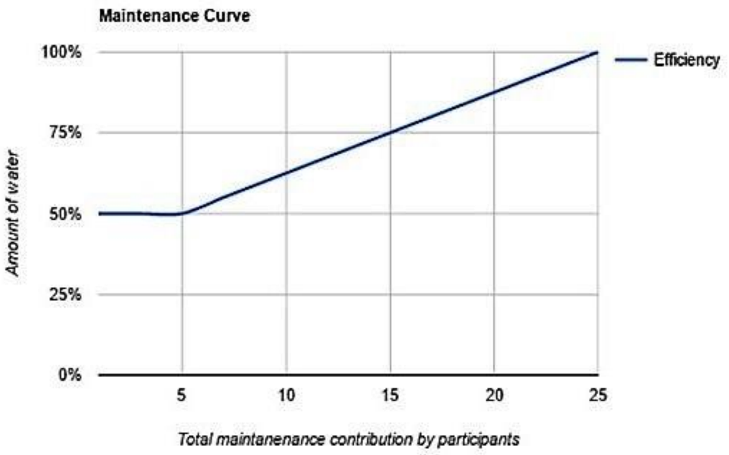

Figure B. Maintenance curve

Supplementary Information for

Relational Quality and Uncertainty in Irrigation Water Management: an exploratory lab experiment

Supplementary S7. Tables

SATISFACTION	Results	Payoff
Structured 1 to 1	-0.506	-0.742
	(1.095)	(0.942)
Unlimited 1 to 1	1.532***	0.872***
	(0.581)	(0.261)
2. Position	0.544	0.882
	(0.960)	(0.737)
3. Position	-0.205	0.461
	(0.774)	(0.744)
4. Position	-0.135	-0.136
	(0.835)	(0.664)
5. Position	-1.158	-2.225**
	(0.861)	(0.879)
N	65	65
chi2	15.348	36.766
p	0.018	0.000

Table A. Estimations of ordered logit regressions by position and communication process

OPINION ABOUT OTHERS	Trust	Fair	Cooperative	Non envious	Non competitive	Understanding	Caring	Non selfish
Structured 1 to 1	-0.072	0.443	0.485	0.593	-0.698	0.848	-0.040	0.403
	(0.839)	(0.777)	(0.666)	(0.762)	(0.469)	(0.608)	(0.630)	(0.832)
Unlimited 1 to 1	1.228	1.402	1.763***	0.493	-0.358	1.740***	0.834	0.996
	(0.923)	(0.906)	(0.525)	(0.557)	(0.560)	(0.600)	(0.511)	(0.664)
2. Position	2.148***	1.639***	1.273*	-0.324	0.370	0.260	1.369***	0.934**
	(0.776)	(0.464)	(0.714)	(0.824)	(0.686)	(0.581)	(0.516)	(0.427)
3. Position	1.817***	0.597	0.756	-0.194	0.669	-0.251	1.350*	0.570
	(0.672)	(0.607)	(0.860)	(0.706)	(0.923)	(0.732)	(0.785)	(0.663)
4. Position	1.124**	0.171	0.330	0.931	0.865	-0.350	0.522	0.581
	(0.546)	(0.480)	(0.379)	(0.849)	(0.809)	(0.851)	(0.752)	(0.649)
5. Position	0.669	-0.919	-0.436	-0.616	-0.430	-1.816**	-0.178	-0.263
	(0.823)	(0.614)	(0.784)	(0.918)	(0.766)	(0.764)	(0.830)	(0.802)
N	65	65	65	62	63	63	62	65
chi2	38.100	18.497	16.953	6.115	8.080	18.584	12.845	7.700
p	0.000	0.005	0.009	0.410	0.232	0.005	0.046	0.261

Standard errors in parentheses, * $p < 0.10$, ** $p < 0.05$, *** $p < 0.01$

Table B. Estimations of ordered logit regressions by position and communication process

	T0			T1			T2		
	P1	P3	Diff.	P1	P3	Diff.	P1	P3	Diff.
Satisfaction	1.880	2.850	+0.970	2.500	3.700	+1.200	1.675	2.211	+0.536
Communication	-	3.700	+3.700	3.517	3.917	+0.400	2.483	3.000	+0.517
Uncertainty	3.420	2.800	-0.620	2.962	2.663	-0.299	3.525	3.579	+0.054
Opinion about others	2.685	3.550	+0.865	3.288	3.717	+0.429	2.917	3.096	+0.179

Table C. Answers to questionnaire. Averages per treatment and phase, and their differences

UNCERTAINTY	Resource			Others' extraction			Others' investment			Stop the round		
Phase	1	3	Diff.	1	3	Diff.	1	3	Diff.	1	3	Diff.
T0	3.36	3.04	-0.32	3.56	2.52	-1.04	3.40	2.68	-0.76	3.36	2.96	-0.40
T1	3.10	2.80	-0.30	3.45	2.80	-0.65	2.40	2.90	+0.50	2.90	2.15	-0.75
T2	3.20	3.26	-0.06	3.65	3.84	-0.19	3.45	3.68	+0.23	3.80	3.53	-0.27

Table D. Average ratings of uncertainty items in the two phases, and their differences

Supplementary Information for

Relational Quality and Uncertainty in Irrigation Water Management: an exploratory lab experiment

Supplementary Answers S8. Answers to average gain disclosure

When asked if knowing average gain changed the opinion held about other players and the course of action taken, head-enders in T2 gave a negative answer or reported having not paid much attention to it (e.g., ¨No¨, ¨Not really¨, ¨I did not see this information¨). Tail-enders instead, reported being influenced by it, making them aware of the inequalities in water use and gains, not necessarily inflicting a worsening of relationships, or decreasing investments in maintenance, using the ultimatum, or just doing nothing (e.g., ¨One of the two first won more than double compared to the other, we put an end to all rounds after that¨, ¨Investment in the canal adjusted¨, ¨Little, because I knew that the first does not really respected our "agreement", but I could not do anything about being the last to pass¨, ¨Yes, for stopping when it was unfair¨).

In T1, several players (both head and tail –enders) indicated that average gain had no effect, or gave no answer about it. Other few however, reported being influenced by it (e.g., ¨Yes, I felt guilty taking a lot and leave little for others¨ (head-ender), ¨Yes, I pass more water in the next round¨ (head-ender), ¨Yes, we first determined the amount that everyone should keep and it worked well¨ (tail-ender), ¨Yes, I have seen a very unequal distribution¨(tail-ender), ¨Yes, I put less in maintenance since I did not pass the units supplied¨ (tail-ender), ¨Yes directly (tail-ender)¨), changing their behaviour by either passing more or less water, lowering their investments, or setting new management deals, but the majority taking no concrete action. Differently than T2, players did not report ultimatum use. A closer look at the content of the verbal exchange among players during P2 and P3, showed that their conversations focused on convincing others to share and invest, without addressing the inequality existing among them. Indicating that they would expect to amend the other players through persuasive dialogue.
